# Dual Roles of Lactate in EGFR-TKI-Resistant Lung Cancer by Targeting GPR81 and MCT1

**DOI:** 10.1155/2022/3425841

**Published:** 2022-12-12

**Authors:** Ruishuang Ma, Xin Li, Shengping Gong, Xiaoqin Ge, Ting Zhu, Xiaoxu Ge, Lijuan Weng, Qingsong Tao, Jianxin Guo

**Affiliations:** ^1^Department of Radiotherapy and Chemotherapy, Ningbo First Hospital, Ningbo, China; ^2^Central Laboratory of the Medical Research Center, Ningbo First Hospital, Ningbo, China; ^3^Department of Respiratory Medical Oncology, Harbin Medical University Cancer Hospital, Harbin, China

## Abstract

Lactate is critical in modeling tumor microenvironment causing chemotherapy resistance; however, the role of lactate in tyrosine kinase inhibitor (TKI) resistance has not been fully known. The aim of this study was to evaluate whether lactate could mediate TKI resistance through GPR81 and MCT1 in non-small-cell lung cancer (NSCLC). Here, we showed that lactate enhanced the cell viability and restrained erlotinib-induced apoptosis in PC9 and HCC827 cells. GPR81 and AKT expression were significantly increased with the addition of lactate, and siGPR81 reduced AKT expression resulting in a raised apoptosis rate with erlotinib treatment. Furthermore, we found that lactate also promoted MCT1 exposure, and inhibiting MCT1 with AZD3965 markedly impaired the glycolytic capacity. A significant increase of GPR81 and MCT1 expression was observed in insensitive tissues compared with sensitive ones by immunostaining in NSCLC patients. Our results indicate that lactate adopts dual strategies to promote TKI resistance in NSCLC, not only activating AKT signaling by GPR81, but also giving energy supply through MCT1-mediated input. Targeting GPR81 and MCT1 may provide new therapeutic modalities for TKI resistance in NSCLC.

## 1. Introduction

In the process of tumor growth, excessive tissue proliferation would cause severe local hypoxia and imbalance of energy metabolism. Tumor cells adapt themselves to ischemia and hypoxia, and thus, glycolytic metabolism are obviously active, resulting in a large amount of lactate (called the Warburg effect) [1–5]. In order to prevent intracellular lactate accumulation from poisoning, tumor cells transport lactate to the outside carrying H^+^ ions at the same time, leading to the decrease of microenvironment pH. Lactate, H^+^, pyruvic acid, and other acidic substances synergistically maintain the acidic state of tumor microenvironment (TME) [1].

For many years, lactate has been considered as a waste product of glycolysis. Recent studies have shown that lactate is not only a metabolite to maintain acid microenvironment homeostasis, but also plays an important role in shaping the behavior and phenotype of tumor and tumor-associated cells [6–9]. Lactate is transferred between hypoxic and oxygen-enriched cells through monocarboxylate transporter (MCT1/4), providing energy for tumor cells, which is faster and more efficient than decomposing glucose, providing energy guarantee for proliferation and metabolism [10–12]. Lactate combines with its receptor (G protein coupled receptor 81, GPR81) promoting the growth of pancreatic cancer cells [13]. Reducing GPR81 expression results in enhanced apoptosis and delayed growth [14]. In addition, lactate dehydrogenase (LDH) is a key enzyme regulating lactate synthesis, which is increased in the late stage of lung cancer and other tumors. Compounds targeting LDH (especially LDHA) have been proposed and validated in preclinical models [15].

The high concentration of lactate is a sign of tumor metabolic adaptability, suggesting poor tumor prognosis [16]. Therefore, as a high-energy substrate of tumor cells, lactate can not only affect the epigenetic changes, but also may become a soluble hormone “sensed” by cell membrane receptors, conducting signal transduction between cancer and nonmalignant cells. However, the roles of lactate in tyrosine kinase inhibitor (TKI) resistance have not been fully elucidated. In this study, we hypothesized that lactate could mediate EGFR-TKI resistance by activating downstream AKT pathway via GPR81 or enhancing glycolysis through MCT1. Targeting lactate metabolism, transport, and its signal transduction process will provide new insights for antitumor therapy in lung cancer.

## 2. Materials and Methods

### 2.1. Reagents

RPMI 1640 medium and fetal bovine serum (FBS) were obtained from Gibco (Grand Island, NY, USA). Bovine serum albumin (BSA), dimethyl sulfoxide (DMSO), and cell counting kit-8 (CCK-8) were from Shanghai Dobio Co., Ltd. (Shanghai, China). Human anti-GPR81 (NLS2095) and anti-MCT1 (MAB8275) were from Novus Biologicals (St. Louis, Missouri, USA). Human anti-AKT (A11016) was from ABclonal Technology (Wuhan, China). Apoptosis kit (AP101) was from Multisciences (Lianke) Biotech Co., Ltd. (Hangzhou, China). MCT1 inhibitor AZD3965 (HY-12750) was from MedchemExpress (MCE, China).

### 2.2. Cell Culture and Treatment

Lung adenocarcinoma cell line PC9 and HCC827 were from BeNa Culture Collection (BNCC, China). Cells were cultured in RPMI-1640 medium supplemented with 10% fetal bovine serum, 1% penicillin, and streptomycin at 37°C in a humidified atmosphere of 5% CO_2_ [17]. For the main tests, cells were treated with erlotinib (final concentration 2 *μ*M) for 24 h or pretreated with lactate (10 mM) for 12 h before erlotinib.

### 2.3. Cell Proliferation Assay

CCK-8 kit was used for cell viability test [17]. In brief, suspensions of cell lines (1 × 10^5^/well) were plated in 96-well plates and grown at 37°C. After treatment, the media were removed, and RPMI1640 (90 *μ*L) and CCK-8 (10 *μ*L) were added. The plates were incubated for 3 h in the incubator. The absorbance at 450 nm was measured on an automated reader (SpetraMax iD3).

### 2.4. Flow Cytometry

To quantify GPR81 and MCT1 expression [18], cells were adjusted to 1 × 106 cells/ml in suspension, and incubated with anti-GPR81 or anti-MCT1 (with concentration of 1 : 100) followed by Alexa-488 conjugated secondary antibody for 15 min in the dark at RT. For apoptosis assay, cells were treated with FITC-Annexin V and propidium iodide (PI) according to the manufacturer's instructions. Cells positive with FITC-Annexin V are considered as early apoptosis and with both FITC-Annexin V and PI staining as late apoptosis. Cells were washed and analyzed on a flow cytometer (FACSVia, Becton Dickinson, USA).

### 2.5. Western Blotting

The AKT expression was measured with western blot as previously described [17]. Whole-cell lysates were resolved on a denaturing 10% SDS-PAGE gel and subsequently transferred to polyvinylidene fluoride membranes via semidry transfer. After blocking the membrane at room temperature with 5% skim milk for 1 h, the membrane was incubated overnight at 4°C with anti-AKT (1 : 1000) antibody. After incubation with peroxidase-conjugated secondary antibodies at a dilution of 1 : 2000 for 1 h, the signals were visualized using enhanced chemiluminescence.

### 2.6. Silencing of GPR81

Cells were transiently transfected with siRNA GPR81 (100 nM, GenePharma, Shanghai, China) using Lipofectamine 2000 Transfection Reagent (Life Technology, Grand Island, NY, USA), according to the manufacturer's instruction (reverse transfections Lipofectamine). The cells were also treated with a scrambled siRNA (GenePharma, Shanghai, China) as a negative control. The efficiency of GPR81 silencing was determined by western blot [17].

### 2.7. Immunofluorescence Assays

Cells were seeded on glass coverslips with polylysine in a 24-well culture plate and fixed with 4% paraformaldehyde (PFA) and then blocked (2% BSA). Cells were incubated with primary anti-AKT (1 : 100) and then with secondary antibodies coupled to Cy3 (1 : 50) [17]. Specimens were analyzed on a fluorescence microscope (Leica, DM400B, Wetzlar, Germany).

### 2.8. Extracellular Acidification Rate

The glycolysis assay (extracellular acidification rate, ECAR) was measured with a test kit (Abcam, ab197244). For details, cells were incubated in a 96-well plate at a density of 3–8 × 10^4^ cells/well and 150 *μ*L of respiration buffer were added before the test. Addition of 10 *μ*L of carbonyl cyanide 4-(trifluoromethoxy) phenylhydrazone (FCCP) was as a positive or negative control. Then, 10 *μ*L reconstituted glycolysis assay reagent was added, and then glycolysis assay signal was measured at 1.5 min intervals for ≥120 minutes using excitation and emission wavelengths of Ex/Em = 380/615 nm, respectively, by a microplate reader (SpectraMax iD3, Molecular Devices).

### 2.9. Immunohistochemistry

A total of 30 NSCLC patients (20 TKI sensitive and 10 TKI resistance) were enrolled and each patient signed an informed consent form for medical record review and tissue sample donation. This study was approved by the Ethics Committee of Harbin Medical University and conducted according to all current ethics guidelines. Tissue sections were immersed in MEDTA, bathed in a steamer at 100°C for 15 min, and incubated in methanol containing 0.3% H_2_O_2_ for 15 min. The slides were incubated with GPR81 (1 : 200) and MCT1 (1 : 100) primary antibodies, stained using DAB, and counterstained using haematoxylin. The relative staining intensity was defined as low <25% and high ≥25% [17]. Cells were imaged with a light microscopy (Leica, Germany).

### 2.10. Statistical Analysis

Results are presented as the mean ± SD of three independent experiments. Statistical differences were evaluated by Student's *t*-test or ANOVA as appropriate. The criterion for statistical significance was set at *p* < 0.05.

## 3. Results

### 3.1. Lactate Ameliorates Erlotinib-Induced Apoptosis in Non-Small-Cell Lung Cancer (NSCLC)

**Figure 1:**
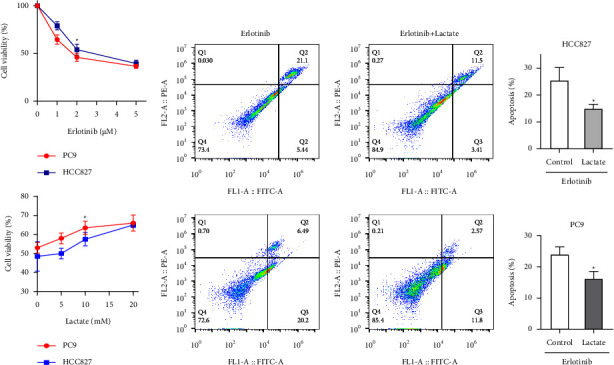
Lactate reduces cytotoxicity induced by erlotinib in lung cancer cells. (a) PC9 and HCC827 cells were pretreated with different concentration of erlotinib, and 2 *μ*M erlotinib could reduce about 50% cell viability of both PC9 and HCC827 cells (upper). CCK-8 assay was performed in the condition of different concentration of lactate (1, 5, 10, and 20 mM) before erlotinib (2 *μ*M). 10 mM lactate could significantly promote the cell viability in both PC9 and HCC827 cells treated with erlotinib (lower). (b) HCC827 and PC9 cells were cultured with 10 mM lactate (right) or untreated (left), and then treated with 2 *μ*M erlotinib. The percentage of apoptosis was measured with flow cytometry. All values are mean ± S.D (*n* ≥ 3). ^*∗*^*P* < 0.05.

The PC9 and HCC827 cells were utilized as EGFR-mutant cell lines of NSCLC, and 2 *μ*M erlotinib could result in a decrease of 50% cell viability (Figure 1(a)). In order to evaluate the effects of lactate on cell viability, different concentration of lactate was added in the medium before erlotinib (2 *μ*M). We found 10 mM lactate significantly promoted cell viability in both PC9 and HCC827 cells (Figure 1(a)). Moreover, 10 mM lactate could reduce the apoptotic percentage of both HCC827 and PC9 cells treated with erlotinib (Figure 1(b)).

### 3.2. Lactate Facilitates Cell Viability by Targeting GPR81 via ATK Signaling

**Figure 2:**
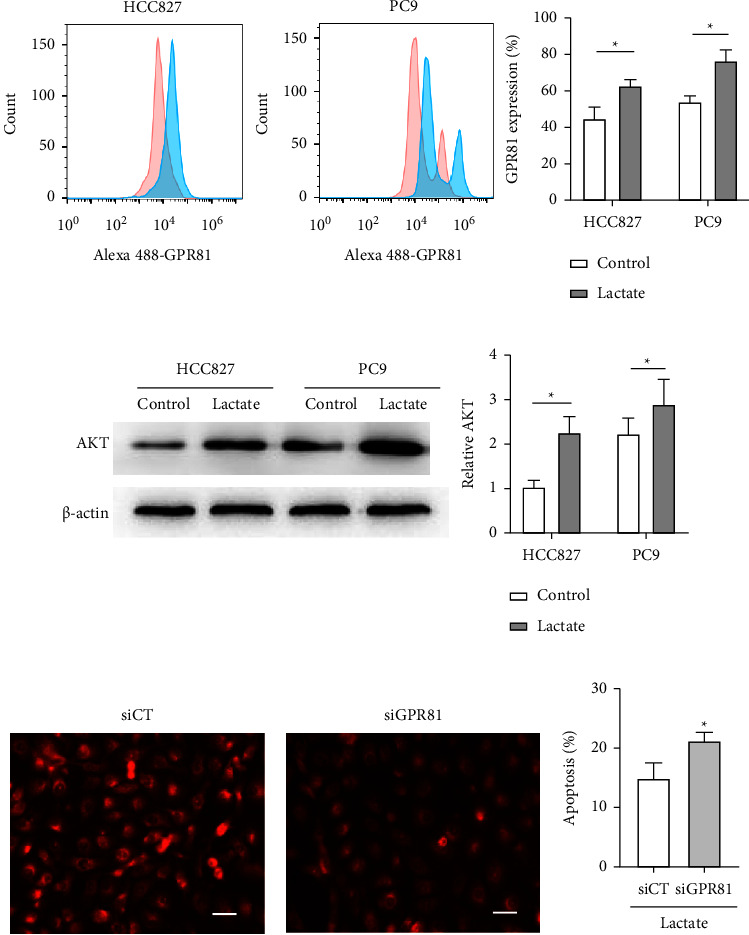
Lactate enhances AKT expression by targeting GPR81-promoting TKI resistance. (a) GPR81 expression was evaluated on HCC827 and PC9 cells by flow cytometry with (blue) or without (red) 10 mM lactate pretreatment. (b) AKT expression of HCC827 and PC9 cells was measured with western blot with or without 10 mM lactate pretreatment. (c) HCC827 cells were cultured with siRNA GPR81 or control (siCT), and then treated with 10 mM lactate. AKT expression was analyzed with immunofluorescence assays (stained red). (d) HCC827 cells were cultured with siRNA GPR81 or control (siCT), and then treated with 10 mM lactate and 2 *μ*M erlotinib. The percentage of apoptosis was measured with flow cytometry. All values are mean ± S.D. (*n* ≥ 3). ^*∗*^*P* < 0.05. Bars represent 20 *μ*m.

HCC827 and PC9 cells were treated with 10 mM lactate, and GPR81 and AKT expression was measured with flow cytometry or western blot separately. Results showed that 10 mM lactate markedly elevated the expression of both GPR81 (Figure 2(a)) and AKT (Figure 2(b)) in both HCC827 and PC9 cells. The immunofluorescence assay showed that silencing GPR81 resulted in sharp decrease of AKT stain in HCC827 cells (Figure 2(c)). Meanwhile, GPR81 shortage by siRNA promoted the apoptosis of HCC827 cells compared with siCT (Figure 2(d)), which may attribute to GPR81-induced AKT downregulation.

### 3.3. Lactate Raises the Rate of Glycolysis through MCT1 Boosting Cell Viability

**Figure 3:**
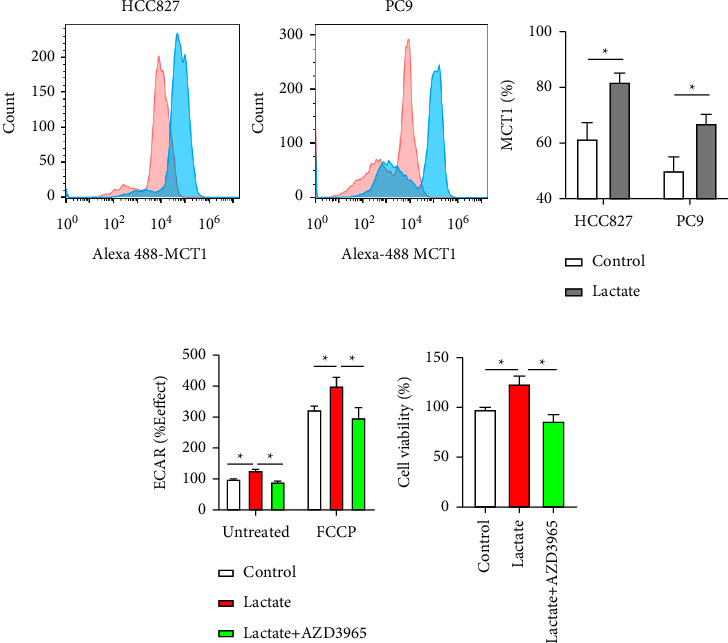
MCT1 increases lactate influx enhancing glycolysis and cell viability. (a) MCT1 expression was evaluated on HCC827 and PC9 cells by flow cytometry with (blue) or without (red) 10 mM lactate pretreatment. (b) Extracellular acidification rate (ECAR) was used to measure glycolysis efficacy in HCC827 cells treated with 10 mM lactate, 10 mM lactate combined with AZD3965 or control. (c) Cell viability was measured in the condition of 10 mM lactate, 10 mM lactate combined with AZD3965 or control. Data are present as mean ± SD. (*n* ≥ 3). ^*∗*^*P* < 0.05.

HCC827 and PC9 cells were treated with 10 mM lactate, and MCT1 expression was measured with flow cytometry. We found increased MCT1 exposure on HCC827 and PC9 cells after lactate addition (Figure 3(a)). Furthermore, the rate of glycolysis was analyzed with ECAR and it showed that AZD3965 (MCT1 inhibitor) significantly blocked glycolysis (Figure 3(b)) and cell viability (Figure 3(c)) in HCC827 cells.

### 3.4. The Expression of GPR81 and MCT1 in NSCLC Patients

**Figure 4:**
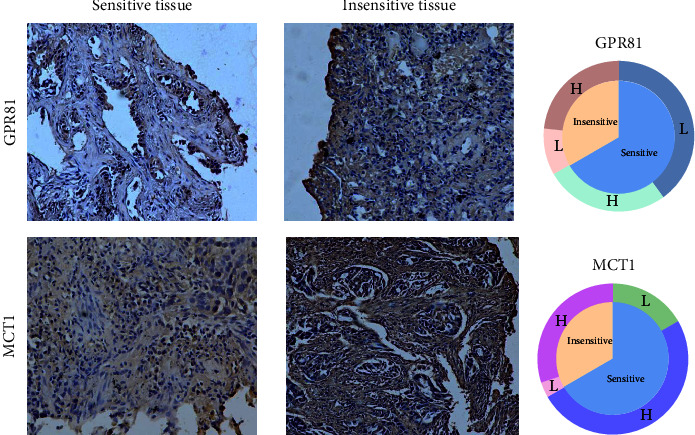
GPR81 and MCT1 in patients of NSCLC. (a) Representative immunostaining of GPR81 and MCT1 expression in sensitive tissues (left) and insensitive tissues (right). Magnification, ×100 (upper), and ×400 (below). (b) The proportion of low/high GPR81 and MCT1 in TKI sensitive (*n* = 20) or resistance (*n* = 10) patients. *L*: low; *H*: high.

**Figure 5:**
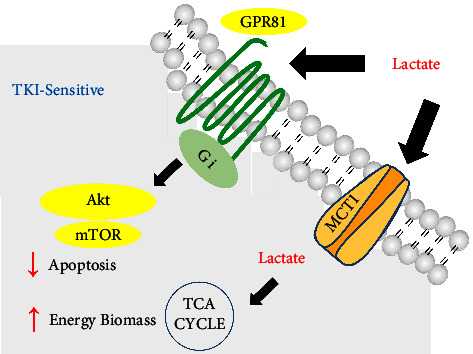
The diagrammatic mechanism of lactate assisting TKI resistance through GPR81 and MCT1. In brief, lactate could adopt dual strategies to promote TKI resistance in NSCLC: on one hand, the combination of lactate and its receptor GPR81 activates the downstream AKT signaling that reduces the apoptosis rate; on the other hand, imported lactate through MCT1 acts as a high-energy substrate for the metabolic biomass. How clever lactate is!

A total of 30 tissue samples from patients with EGFR-mutation were collected including 20 TKI-sensitive and 10 TKI-resistant samples. The immunohistochemistry images showed the GPR81 and MCT1 expression in sensitive tissues and insensitive ones (Figure 4(a)). The percentage of high or low of GPR81 and MCT1 expression was also evaluated (Figure 4(b)), indicated that TKI-resistant patients may have more GPR81 and MCT1 expression compared with TKI-sensitive patients. The diagrammatic mechanism of lactate assisting TKI resistance through GPR81 and MCT1 was shown in Figure 5. In brief, lactate could adopt dual strategies to promote TKI resistance in NSCLC: on one hand, the combination of lactate and its receptor GPR81 activates the downstream AKT signaling that reduces the apoptosis rate; on the other hand, imported lactate through MCT1 acts as high-energy substrate for the metabolic biomass. How clever lactate is!

## 4. Discussion

When people are sick, medicine and nutrition support are essential for the guarantee of rapid rehabilitation. By the same token, tumor cells also need protection in therapeutic conditions like drugs and radiation. In this study, we for the first time showed that lactate not only functions as “medicine” to activate AKT signaling for the sake of survival, but provides “nutrition” as high-energy substrate for prompt metabolic capacity. Targeted drug resistance is a tricky problem in clinical treatment, and thus, we speculate that lactate may be an accessory contributing to the resistance of EGFR-TKI in NSCLC.

As an important extracellular signaling molecule, lactate has gained more and more attention especially in modeling TME. Lactate together with its receptor GPR81 is reported in lipolysis by activating cAMP [19], and recently Wu et al. showed that lactate could enhance the effect of the parathyroid hormone on osteoblast differentiation via GPR81-PKC-Akt signaling [20]. Here, we found that lactate rescued the cell viability in case of erlotinib in NSCLC cells, where activity would be weakened when GPR81 was knocked down. Moreover, by targeting GPR81, lactate could promote AKT protein expression, which is a key character in EGFR downstream pathway. Taken together, we indicate that lactate ameliorates TKI-induced apoptosis through a bypass-activated resistance pathway.

Monocarboxylate transporters are monitor gates for lactate fluxes, and their roles are predominant in metabolic reprogramming as well as therapeutic resistance. Extracellular lactate uptake via MCTs not only brings “digestible” carbon source to cancer cells for energy replenishment, but also controls histone modification via lactylation in various biological functions [21, 22]. Targeting MCTs especially MCT1 by small molecular inhibitors has been demonstrated to inhibit tumor aggressiveness, and MCT1 inhibitors are being in the advanced development phase [23]. In this study, MCT1 inhibitor AZD3965 was found to attenuate the metabolic benefits brought by lactate, which could give assistance to the erlotinib's effect.

Numerous histone modifications, including acetylation, methylation, and crotonylation, have been documented before the 2019 discovery of histone lactylation. Recent findings by Zhao et al. state that lactate contributed to epigenetic regulation of genes by lactylating histone lysine residues and that lactate was found to be a precursor to histone lysine lactylation (Kla), which stimulated gene transcription from chromatin [24, 25]. Histone lactylation is first reported to promote homeostasis of macrophages during infection, and more and more studies have focused its role in cancer. Not only dose histone lactylation regulate oncogenes in the process of tumorigenesis of cancer cells, but facilitates immunosuppression of tumor-infiltrating myeloid cells and regulatory *T* cells [26–28]. A recent work by Jiang et al. showed that lactate induces transcriptional activation of HIF1a in lung cancer, which in turn activates transcription of genes that facilitate glucose uptake and glycolysis resulting in lactate generation with a sort of positive feedback [29]. However, the role of histone lactylation in therapeutic resistance is largely unknown, and future studies are needed to provide new insight with a boarder perspective.

In conclusion, we suggest that lactate adopts dual strategies to promote TKI resistance in NSCLC. On one hand, lactate activates AKT signaling by targeting GPR81, and on the other hand, MCT1-assissted lactate input meets the energy need. With the in-depth study of lactylation, it is believed that more functions of lactate will be uncovered and the mystery of lactate and its partners will be gradually unveiled in therapeutic resistance.

## Data Availability

The original contributions presented in the study are included in the article/Supplementary Material, further inquiries can be directed to the corresponding authors.
